# IL-17/miR-192/IL-17Rs Regulatory Feedback Loop Facilitates Multiple Myeloma Progression

**DOI:** 10.1371/journal.pone.0114647

**Published:** 2014-12-09

**Authors:** Yuanyuan Sun, Jing Pan, Shudan Mao, Jieping Jin

**Affiliations:** Department of Hematology, the First Affiliated Hospital of Liaoning Medical University, Jinzhou, Liaoning, China; Thomas Jefferson University, United States of America

## Abstract

Multiple myeloma (MM) is a clonal plasma cell disorder which constitutes the second most common hematological malignancy, and remains an incurable tumor with poor survival. Recently, interleukin-17 (IL-17), produced locally in the tumor microenvironment, has been reported to play a crucial role in tumor immunity. In this study, we determined that exposure of MM cells to IL-17 had various promotive influences on different aspects of tumor progression. IL-17 significantly induced cell proliferation, inhibited cellular apoptosis, repressed cell adhesion to fibronectin and collagen I, and facilitated cell migration. Exposure to IL-17 also resulted in epithelial-mesenchymal transition (EMT), as evidenced by repression of the epithelial marker E-cadherin, and induction of the mesenchymal marker Vimentin, and EMT transcription factors Snail and Slug. Further experiments showed that IL-17 activated the oncogenic p65 transcription factor, which directly repressed the miR-192 gene via binding to the miR-192 promoter. Loss of miR-192 in MM cells can mimic the effects of IL-17, and was required for the above oncogenic effects of IL-17 on MM. Furthermore, we found that miR-192, and its homologous miR-215 directly targeted the 3′-untranslated regions of IL-17Rs, including IL-17RA and RE mRNA. By examining bone marrow specimens derived from MM patients, a negative correlation between miR-192 expression and IL-17 or IL-17RA expression was observed. Also, IL-17 was negatively correlated with E-cadherin and positively with Vimentin. Taken together, our study provides evidence that the IL-17/miR-192/IL-17Rs regulatory feedback loop is manifest in MM and might represent a promising and efficient prognostic marker and therapeutic target for MM.

## Introduction

Cancer is one of the major health problems in the Western world, with very poor prognosis and high possibility of metastasis [1]. As the second most common hematological cancer in the USA, multiple myeloma (MM) is characterized by a monoclonal proliferation of malignant plasma cells and develops from a complicated network composed of various molecular processes [2]. During progression to metastasis, the interactions between myeloma cells with the components of their microenvironment are thought to play an important role in driving these cells malignant [3]. These interactions are critical to multiple stages in tumorigenesis, from initial homing to the hematopoietic stem cells niche, escape from normal immune suppression, and resistance to chemotherapeutic treatments, to support of tumor growth and development of cancer-induced complications [4]. Thus, tempering the response of host microenvironment to myeloma is of great therapeutic value.

Among the multiple host microenvironment factors, the pro-inflammatory interleukin-17 (IL-17), produced by a lineage of CD4^+^ T helper cells (TH17 cells), has been shown to be involved in several chronic pathologies, such as inflammation, autoimmune disease, and tumors [5]–[7]. The levels of TH17 cells are significantly increased in multiple mouse and human tumors including head and neck cancer, melanoma, prostate cancer, sarcoma, ovarian cancer, renal cancer, and pancreatic cancer [5]. Consistently, the IL-17 polymorphisms are associated with oral, gastric, esophageal, ovarian and breast cancer [8]. Univariate and multivariate analysis reveals that IL-17 is an independent prognostic factor for overall survival of patients with colorectal cancer, and TH17 cells may facilitate development of cancer by fostering angiogenesis via promoting VEGF production from cancer cells [9]. Increase in circulating levels of IL-17 together with IL-6, VEGF and TNF-α contributes to the increased breast-cancer-associated lung metastasis and bone metastasis in pro-arthritic and arthritic mice. And treatment with anti-IL17^+^ celecoxib completely abrogates the development of metastasis and significantly reduces the primary tumor burden [10]. In MM, significant elevated levels of IL-17 are found in bone marrow and peripheral blood of the newly diagnosed patients and relapsed patients; moreover, the IL-17 levels in patients with stage II and stage III tumor are higher compared to those of stage I [11]. Proportion of TH17 cells is also increased in patient with MM, and the elevated level correlates with clinical tumor stage. After myeloma peripheral blood mononuclear cells (PBMCs) are TH17 polarized, the induced IL-17 significantly promotes myeloma cell growth and colony formation via IL-17 receptor, adhesion to bone marrow stromal cells, as well as cell growth in murine xenograft model of MM [12]. However, the underlying mechanisms by which IL-17 and its related genes regulate human MM development and progression remain largely unknown.

MicroRNAs (miRNAs) are endogenous small non-coding RNAs which negatively regulate gene expression either by translational repression, or target mRNA degradation via binding target mRNAs through the 3′-untranslated region (3′-UTR) [13]. miRNAs have important regulatory functions in biological processes which represent the hallmarks of cancer, such as proliferation, apoptosis, invasion, and metastasis [14]. Abnormal expressions of many miRNAs in cancer have been reported. Among these miRNAs, miR-192 has been demonstrated to be significantly downregulated in multiple cancer types and play a role of tumor suppressor. For example, miR-192 was markedly decreased in metastatic renal cell carcinoma and restoration of its expression decreased cell migration and invasion in renal cell carcinoma by targeting ZEB2, MDM2 and TYMS [15]. In colorectal cancer, overexpression of miR-192 significantly influenced 5-fluorouracil resistance and reduced cell proliferation by targeting cell cycle progression [16]. It was also reported that in MM, miR-192 can be transcriptionally activated by p53 and then target the IGF pathway to prevent migration of plasma cells into bone marrow [17]. These data all indicate miR-192 may serve as a molecular target for tumors, including MM.

In this study, we aimed to investigate effect of IL-17 on growth and metastasis of MM cells, and the intricate networks among molecules which control these processes. We found IL-17 could induce proliferation, migration and epithelial-mesenchymal transition (EMT) of MM cells, as well as inhibit cellular apoptosis. A novel feedback circuit established by IL-17 mediated suppression of miR-192 and miR-192 regulation of IL-17 Receptor (IL-17Rs) was involved in these activities. Our findings provide further evidence for the implication of pro-inflammatory cytokine and dysregulated miRNAs in MM, and suggest particular therapeutic impacts of IL-17 and miR-192.

## Materials and Methods

### Tissue samples

Bone marrow samples were collected from twenty-two patients with MM (12 male and 10 female) who were hospitalized at Department of Hematology, the First Affiliated Hospital of Liaoning Medical University from 2008 to 2013. For the use of clinical materials for research purposes, prior approval was obtained from the Medical Ethics Committee of Liaoning Medical University (#LMU 5JZ-1013204). The study was conducted according to the principles expressed in the Declaration of Helsinki. All samples were collected and analyzed with prior written, informed consent of the patients. The study was carried out in accordance with the institutional ethical guidelines and the use of human bone marrow tissues was approved by the Medical Ethics Committee of Liaoning Medical University (#LMU 5JZ-1013204). All samples were freshly frozen in liquid nitrogen, and then stored at −80°C for further use.

### Cell culture, reagents and cell transfection

Human MM cell lines MM1S, MM1R and H929 were obtained from American Type Culture Collection and routinely maintained in RPMI-1640 medium supplemented with 10% fetal bovine serum in a 37°C humidified atmosphere of 5% CO_2_. IL-17A was dissolved in water and used at indicated final concentration for 12 h. Overexpression of miRNAs in cells was obtained by transfection with miR-192 mimics: 5′-CUGACCUAUGAAUUGACAGCC or miR-215 mimics: 5′-AUGACCUAUGAAUUGACAGAC using Lipofectamine2000. Knockdown of miR-192 and p65 was performed using miR-192 inhibitor (5′-CUGCCAAUUCCAUAGGUCACAG) or p65 siRNA (5′-GCCCUAUCCCUUUACGUCA) respectively. For cells received combined treatment, cells were treated with miR-192 mimics transfection first, followed by IL-17A stimulation at 12 h before the endpoint of the indicated assay.

### RNA extraction and quantitative real-time PCR analysis

Total RNA was extracted from tissues or cells using Trizol reagent or miRNeasy Mini Kit according to the manufactures’ instructions. For miRNA expression, total RNA was reverse-transcribed using the Taqman miRNA reverse transcription kit and real-time PCR was performed using TaqMan Universal Master Mix II. For mRNA expressions, total RNA was reverse-transcribed using RT reagent Kit and real-time PCR was carried out using SYBR green PCR master mix. miRNA and mRNA expressions were normalized using detection of U44 snRNA or GAPDH respectively. Gene expression was measured in triplicate and data were processed using 2^−ΔΔ^CT method and normalized to control.

### Protein extraction and western blot analysis

Cells were lysed in RIPA buffer (50 mM Tris/HCl, pH 8.0, 250 mM NaCl, 1% NP40, 0.5% [w/v] sodium deoxycholate, 0.1% sodium dodecylsulfate) with protease inhibitors. Lysates were centrifuged at 20,000 g for 30 min at 4°C. Protein was subjected to a 10% SDS-acrylamide gel, transferred onto PVDF membrane and blotted using indicated primary antibodies. Signals from HRP-conjugated secondary antibodies were generated by ECL Substrates. GAPDH served as the loading control.

### Chromatin Immunoprecipitation (ChIP) analysis

Cells were grown to 80% confluency and cross-linked with 1% formaldehyde at 37°C for 10 minutes. Cells were then washed with ice-cold PBS and resuspended in 0.5 mL of lysis buffer containing 1% sodium dodecyl sulphate, 10 mM EDTA, 50 mM Tris-HCl, 1× protease inhibitor cocktail and sonicated to fragments of 200–500 bp. Antibodies against p65 or rabbit IgG control were added to each aliquot of pre-cleared chromatin and incubated overnight. Protein A and G-agarose beads were added and incubated for 2 h at 4°C. After reversing the cross-linking, DNA was extracted with phenol-chloroform and used for PCR assay. Primers used for ChIP assay: Binding site 1: forward 5′-ACAGAGGGTTCAAGGTTTGG-3′ and reverse 5′- TCATCTCCCGCAGGTTCTT-3′; Binding site 2: forward 5′- CATGTGGAACCTGCTGAATG-3′ and reverse 5′- CCACTTCCTCCCACTCTTCC-3′. Primers for acetylcholine receptor were used as control.

### Proliferation assay

Cell proliferation rates were evaluated using a WST-8 assay. Cells were seeded in a 96-well culture plate at a density of 4,000 cells, treated with IL-17, miR-192 inhibitor or mimics as indicated. WST-8 reagent (10 µl) was added to each well at 1 h before the endpoint of incubation. OD450 nm value in each well was determined by a microplate reader.

### Trypan Blue exclusion viable cell assay

Cells treated as described above were trypsinized and resuspended in equal volumes of medium and stained with 0.4% trypan blue solution. Cells were then counted using a haemocytometer. Viable cells number was assessed based on exclusion of trypan blue dye and cells that took up trypan blue were counted as dead cells.

### Apoptosis assay

Apoptosis assays were performed using Annexin-V apoptosis detection kit. Briefly, cells were collected, washed with PBS and resuspended in binding buffer containing Annexin V-FITC and propidium iodide (PI). After 15 min of incubation at room temperature, samples were analyzed on a FACSCalibur flow cytometer to determine rate of apoptosis.

### TUNEL staining

Visualization of DNA fragmentation was performed using the fluorometric TUNEL system for cellular apoptosis according to the manufacturer’s instructions. After centrifugation, cells were fixed in 4% paraformaldehyde at room temperature for 15 min, and incubated with fluorescein-conjugated TdT enzyme at 37°C for 1 h in dark. Cells were then mounted on glass slides, and examined with a fluorescence microscope.

### Immunofluorescence assay

Cells were fixed in 4% paraformaldehyde for 15 min at room temperature, penetrated with 0.5% Triton X-100 for 5 min, and blocked with 5% BSA. Then cells were incubated with PE-conjugated anti-Ki-67 antibody for 1 h on ice. Cells with PE- conjugated normal mouse IgG1 were used as the isotype control. After three washes, cells were analyzed on a FACSCalibur flow cytometer.

### Adhesion assay

Untreated 96-well flat-bottomed tissue culture plates were coated with 2.5 g/cm^2^ of fibronectin or 3.67 g/L of rat-tail collagen I overnight at 4°C. Cells were seeded at a density of 4,000 cells per well. After incubation for 60 min, cells were treated with percoll flotation medium and percoll fixative for 15 min, stained with 0.5% crystal violet, and allowed to dry overnight. Cell attachment was determined by a microplate reader at OD590 nm on the next day, once each well was solubilized with Sorenson solution.

### Migration assay

The migratory potential was evaluated using Costar 24-well plates containing 8 µm pore size polycarbonate filter inserts. Briefly, 2×10^5^ cells were resuspended in serum-free medium and added to the upper inserts; 500 µl media containing 10% FBS with or without IL-17 were added to the matched lower chamber. After 48 h, non-migrating cells were removed using a cotton swab, and the underside of the insert was stained with crystal violet. Cells migrated to media in the lower chamber were also collected, stained with trypan blue and counted. Migration was calculated as total cells including those on the lower membrane surface and those in the lower supernatant.

### Luciferase reporter assay

The fragment of 3′-UTRs of IL-17Rs (IL-17RA, IL-17RC, and IL-17RE) containing miRNAs binding site were PCR amplified and then inserted into pGL3-basic vector. Target site mutations were generated using the PCR products with the appropriate primers containing point substitutions. The sequences were verified by DNA sequencing. HEK293T cells were plated in 96-well plates and co-transfected with reporter plasmid with miRNA mimics or control miRNA. At 48 h after transfection, luciferase activity was detected using a dual-luciferase reporter assay system and normalized to Renilla activity.

### Statistical analysis

All data from 3 independent experiments were expressed as mean ± SD and processed using the SPSS 13.0 software. A P-value of <0.05 was considered to indicate a statistically significant result. The differences among the groups were estimated by Student’s t-test or one-way ANOVA. The Mann-Whitney U test and Spearman’s correlation analyses were used to analyze the relationship between miRNA expression and mRNA expression.

## Results

### IL-17 leads to increased growth and metastatic properties of MM cells

**Figure 1:**
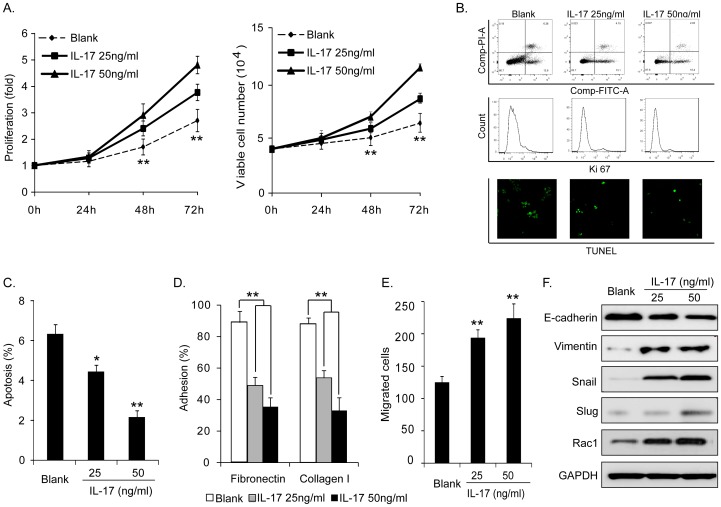
Effects of IL-17 on proliferation, apoptosis, adhesion, migration and EMT properties of MM1S cells. (A) IL-17 induced cell proliferation was analyzed by WST-8 assays (left) and Trypan Blue exclusion viable cell assay (right). (B) IL-17 inhibited cell apoptosis determined by Annexin-V binding assay (top), Ki-67 staining (middle), and TUNEL assay (bottom). (C) Canonical histogram of apoptotic rate characterized by Annexin-FITC positive cells was shown. (D) IL-17 decreased cell adhesion to fibronectin and collagen I. (E) IL-17 increased cell migration. (F) IL-17 induced EMT and Rac1 expression of cells. Epithelial marker E-cadherin, mesenchymal marker Vimentin, EMT transcription factors Snail and Slug, and Rac1 expression were detected by western blot analysis. Note all the effects induced by IL-17 were in a dose-dependent manner. (**P<0.01, *P<0.05, Figure is representative of 3 experiments with similar results.).

To determine the effects of IL-17 on biological function of MM cells, we treated the human MM cell line MM1S with recombinant IL-17A. As expected, treatment of MM1S cells with IL-17 significantly induced cell proliferation in a dose-dependent manner, determined by WST-8 proliferation assay (Fig. 1A left) and Trypan Blue exclusion viable cell assay (Fig. 1A right). Next, we determined the rate of cellular apoptosis by Annexin-V binding assay which depends on the loss of the cell membrane’s phospholipid asymmetry (Fig. 1B top and Fig. 1C), and TUNEL assay which depends on the presence of DNA fragmentation (Fig. 1B bottom). Moreover, because Ki-67 antigen is present in proliferating cells throughout the cell cycle, we also stained cells with monoclonal Ki-67 antibody to evaluate the proportion of proliferating cells (Fig. 1B middle). Although difference of Ki-67 staining in cells with and without IL-17 treatment was slight, it was still notable that cellular apoptosis was significantly lower upon stimulation with IL-17 indicated by Annexin-V binding assay and TUNEL assay (Fig. 1B and 1C). Cellular adhesion was measured using two different extracellular matrixes (ECM), fibronectin and collagen I. IL-17 treatment caused decreased cell adhesion to fibronectin and collagen I (Fig. 1D). Cell motility was evaluated by migration assay. As shown in Fig. 1E, compared to the controls, IL-17 treated cells exhibited significant increased migratory ability. IL-17 treatment also resulted in EMT. Western blot showed that the epithelial marker E-cadherin was repressed, and the mesenchymal markers Vimentin was increased; meanwhile, EMT transcription factor Snail and Slug were induced (Fig. 1F). Moreover, IL-17 treatment could upregulate oncogenic Rac1 expression. Taken together, these results showed that MM1S cells undergo an increase in metastatic capacity in response to IL-17 exposure, which is accompanied by increased proliferation, impaired apoptosis and adhesion, enhanced migration and induced EMT.

### IL-17 directly represses miR-192 expression

**Figure 2:**
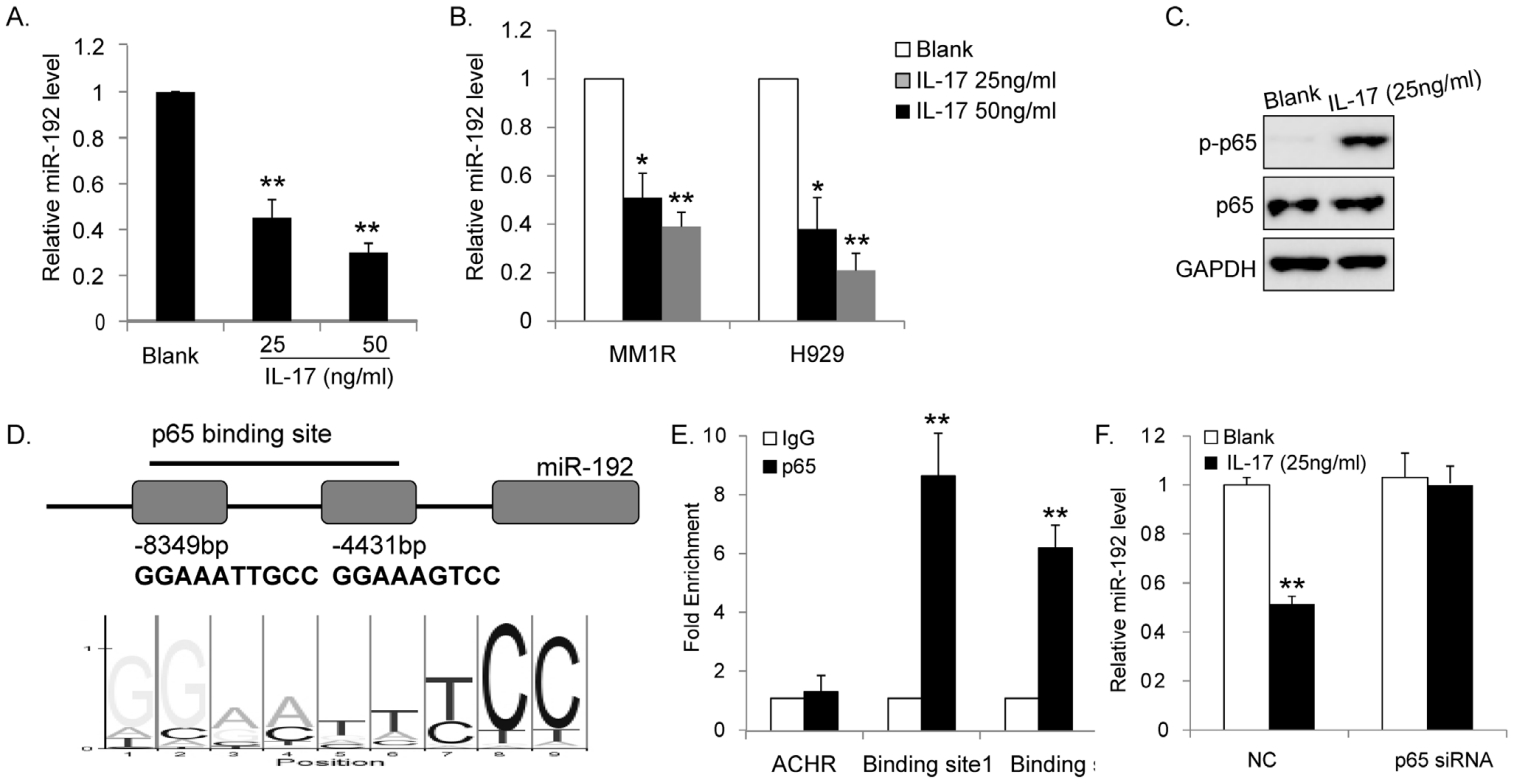
IL-17 directly repressed expression of miR-192. (A) MM1S cells were treated with IL-17 for 12 h and miR-192 expression was determined by qPCR analysis. IL-17 treatment significantly downregulated miR-192 in a dose-dependent manner. (B) IL-17 treatment significantly downregulated miR-192 in MM1R cells and H929 cells. (C) IL-17 treatment activated p65 pathway. (D) Location and sequence of predicted p65-binding sites in the promoter of miR-192 gene. (E) ChIP assay was performed and indicated that p65 could bind to the indicated regions of miR-192 promoter. (F) siRNA-mediated downregulation of p65 prevented the repression of miR-192 after IL-17 treatment. (**P<0.01, *P<0.05, Figure is representative of 3 experiments with similar results.).

We analyzed miR-192 expression in MM1S cells upon IL-17 treatment. After exposure to IL-17 for 12 h, the expression of miR-192 decreased in a dose-dependent manner (Fig. 2A). Expressions of miR-192 were also repressed after exposure of H929 cells and MM1R cells to IL-17 (Fig. 2B). Therefore, this effect is not restricted to MM1S cells, but is presumably a general response of MM cells.

We found IL-17 treatment induced activation of p65 pathway as shown in Fig. 2C. Subsequently, by analyzing the miR-192 promoter region (about 1 kb upstream of the miR-192 stem loop) using the TFSEARCH program [18], we found several potential p65-binding sites that might regulate miR-192. Then we performed ChIP assay and our results showed that p65 could bind to the two indicated regions (Fig. 2D and E). Moreover, siRNA-mediated knockdown of p65 prevented the repression of miR-192 after IL-17 treatment, confirming that p65 mediates the repression of miR-192 observed after IL-17 treatment (Fig. 2F). In summary, IL-17 could directly downregulate miR-192 through activating p65 pathway.

### Loss of miR-192 can mimic the effects of IL-17 on MM cells

**Figure 3:**
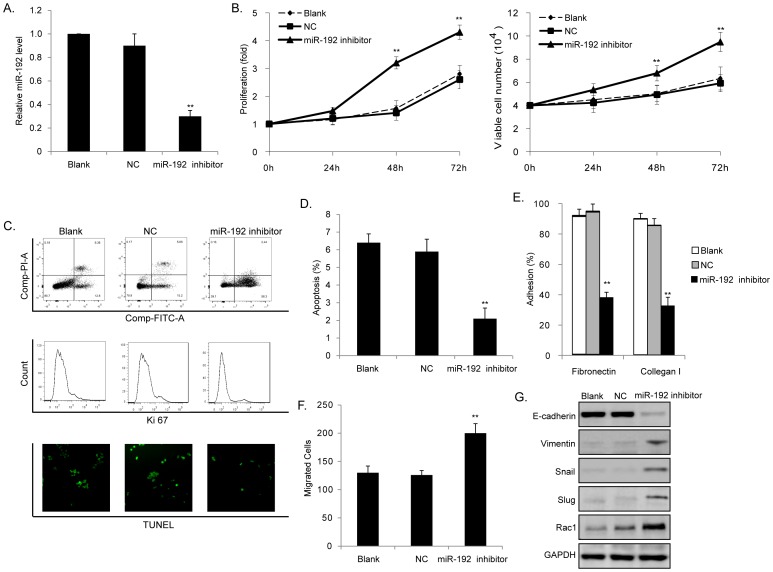
Loss of miR-192 mimicked the effects of IL-17 on MM cells. (A) Downregulation of miR-192 in MM1S cells was induced by transfecting cells with miR-192 inhibitor. (B) Loss of miR-192 induced cell proliferation. (C) Loss of miR-192 repressed cellular apoptosis. (D) Canonical histogram of apoptotic rate characterized by Annexin-FITC positive cells was shown. (E) Loss of miR-192 decreased cell adhesion to fibronectin and collagen I. (F) Loss of miR-192 promoted cell migration. (G) Loss of miR-192 induced EMT and Rac1 expression of cells. (**P<0.01, *P<0.05, Figure is representative of 3 experiments with similar results.).

We downregulated expression of miR-192 in MM1S cells to see whether knock down of miR-192 alone could produce the same effects as treatment of IL-17. Downregulation of miR-192 in MM1S cells was induced by transfecting cells with miR-192 inhibitor (Fig. 3A). Consistent with the effects of IL-17 treatment, loss of miR-192 significantly induced cell proliferation (Fig. 3B), repressed cellular apoptosis (Fig. 3C, D), decreased cell adhesion to fibronectin and collagen I (Fig. 3E), and promoted cell migration as well (Fig. 3F). Moreover, cells with respectively lower expression of miR-192 presented decreased level of E-cadherin, increased levels of Vimentin, and induced Snail, Slug as well as Rac1 expression (Fig. 3G).

### miR-192 plays a crucial role in IL-17-regulated growth and metastatic properties of MM cells

**Figure 4:**
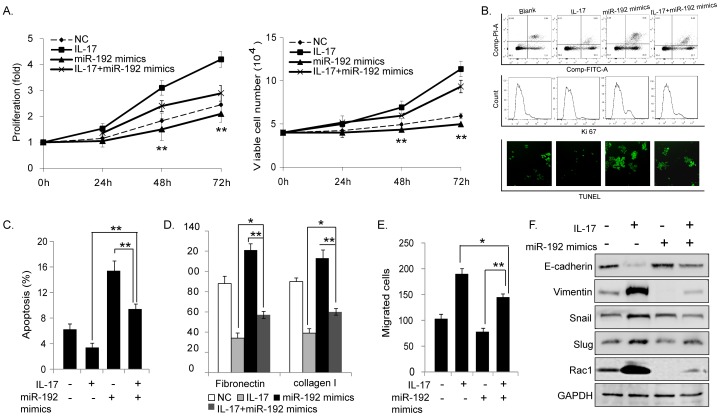
Oncogenic effects of IL-17 on MM cells were mediated by repression of miR-192. Functional effects of IL-17 on MM1S cells with ectopic expression of miR-192 were evaluated. Overexpression of miR-192 significantly inhibited cell proliferation (A), induced cell apoptosis (B and C) and adhesion (D), suppressed cell migration (E) and EMT (F). IL-17 stimulation alleviated the above inhibitory effects of miR-192 compared with treatment with miR-192 mimics transfection alone. (**P<0.01, *P<0.05, Figure is representative of 3 experiments with similar results.).

To further confirm the potential relationship between IL-17 and miR-192, we detected the above biological functions of MM1S cells under the treatment of miR-192 mimics transfection combined with IL-17 stimulation. As shown in Fig. 4, the increased expression of miR-192 significantly inhibited proliferation (Fig. 4A), induced apoptosis (Fig. 4B, C) and adhesion (Fig. 4D), repressed migration (Fig. 4E) and EMT ability (Fig. 4F) of MM cells. Moreover, when cells were treated with miR-192 mimics together with IL-17, the ectopic expression of miR-192 could even block IL-17 induced cancer progression. These results indicated that IL-17 induced growth and metastatic properties of MM cells was mediated by repression of miR-192.

### IL-17Rs, including IL-17RA and IL-17RE are direct targets of miR-192 and miR-215

**Figure 5:**
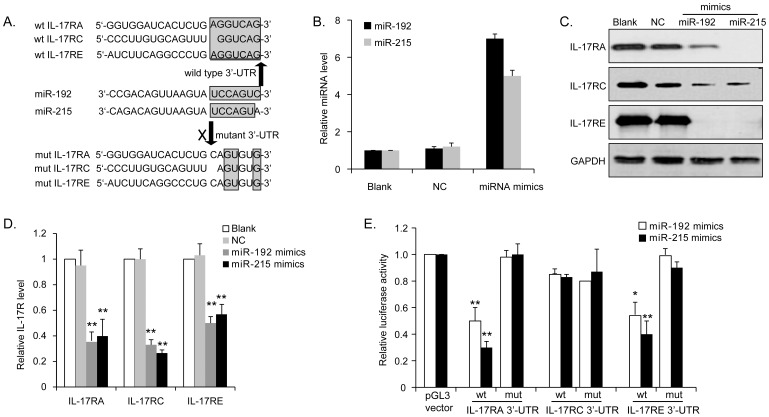
miR-192 directly targets IL-17Rs, including IL-17RA and IL-17RE. (A) The predicted miR-192 and miR-215 binding sites within 3′-UTRs of IL-17RA, IL-17RC and IL-17RE, and their mutated versions by site mutagenesis. Upregulation of miR-192 and miR-215 (B) repressed expressions of IL-17RA, IL-17RC and IL-17RE at both protein (C) and mRNA (D) level. (D)The repression of luciferase activities by 3′-UTRs of IL-17RA and IL-17RE, but not IL-17RC, was dependent on miR-192 and miR-215. Mutated 3′-UTRs of IL-17RA and IL-17RE abrogated miR mediated repression of luciferase activity. (**P<0.01, Figure is representative of 3 experiments with similar results.).

Since miRNAs are often components of feedback loops, we hypothesized that miR-192 itself may target components of the IL-17 signaling pathway. By using the miRNA target prediction database (miRBase, miRNA.org and Target Scan), we proposed that IL-17Rs, including IL-17RA, IL-17RC and IL-17RE were putative targets of miR-192 and miR-215, who shares the exactly same seed sequences as miR-192 (Fig. 5A). Ectopic expressions of miR-192 and miR-215 in MM1S cells (Fig. 5B) repressed both protein (Fig. 5C) and mRNA (Fig. 5D) levels of IL-17RA, IL-17RC and IL-17RE. To further investigate if the predicted binding sites of miR-192 and miR-215 to 3-’UTR of IL-17Rs are responsible for this regulation, the IL-17Rs 3-’UTR fragment, containing the wild type or mutant miRNA binding sequences, were cloned into the Renilla luciferase reporter, and co-transfected into HEK293T cells with miRNA mimics or scrambled control. When wt-IL-17RA or wt-IL-17RE vector was introduced into the co-transfection system, the luciferase activities of miRNA mimics transfected cells were significantly reduced compared to control cells. Mutation in the corresponding putative binding site abolished the miRNAs-mediated repression of luciferase activity. But when wt- or mut-IL-17RC was used, no significant change of luciferase activity was observed (Fig. 5E). These results demonstrated that miR-192 and miR-215 could directly target IL-17RA and IL-17RE in MM cells by interaction with the 3′-UTRs.

### The IL-17/miR-192/IL-17Rs feedback loop is characteristic for MM

**Figure 6:**
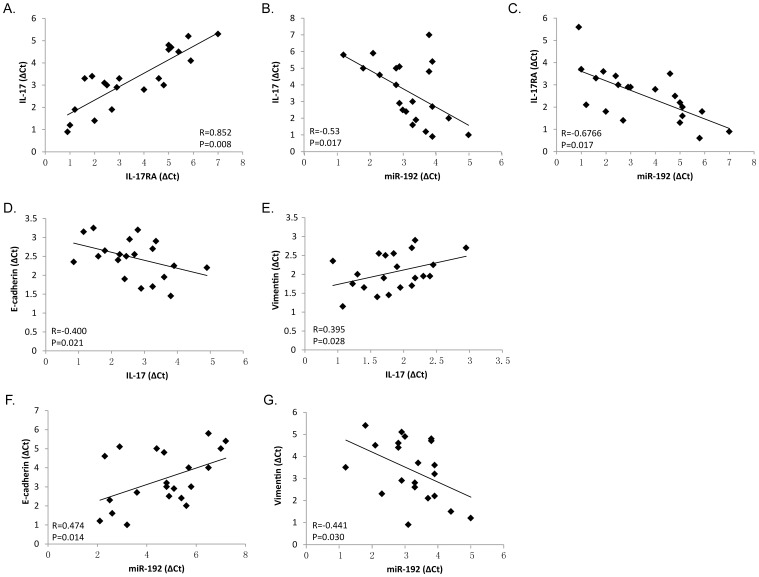
The IL-17/miR-192/IL-17Rs feedback loop is characteristic for MM. Correlative analysis of the indicated mRNAs and miR-192 in bone marrow tissues of patients with MM was detected by qPCR. Spearman correlation coefficient with the respective significance was indicated.

To test whether the regulations described above for MM cell lines are also clinically relevant, we examined bone marrow specimens derived from 22 MM patients. As shown in Fig. 6, a positive association of IL-17 and IL-17RA expression (Fig. 6A) and a negative correlation between the expression of miR-192 and IL-17 levels (Fig. 6B) or IL-17RA levels (Fig. 6C) were observed. Moreover, expression of IL-17 negatively correlated with EMT-associated epithelial marker E-cadherin and positively correlated with mesenchymal maker Vimentin (Fig. 6D, E). Conversely, the expression of miR-192 positively correlated with E-cadherin (Fig. 6F) and negatively with Vimentin (Fig. 6G).

## Discussion

Tumor development and progression have been demonstrated to be supported by chronic inflammatory conditions which develop in the tumor microenvironment and are characterized by various inflammatory factors [19], [20]. Recently, there is growing evidence that IL-17 plays an essential role in the recruitment of inflammatory cells to tumor sites, and inhibition of IL-17 function has been suggested as a very promising therapeutic approach for inflammatory diseases and tumor [5], [10], [21]. In this study, we identified an IL-17-triggered feedback loop which involves miR-192-targeted IL-17Rs, including IL-17RA and IL-17RE, and controls cellular growth and progression in MM. IL-17 significantly facilitated metastatic capacity of MM cells, which is accompanied by increased proliferation, impaired apoptosis and adhesion, enhanced migration and induced EMT. More importantly, IL-17 directly repressed expression and function of miR-192; and miR-192 sequentially targeted IL-17RA and IL-17RE, which binds the ligand IL-17 to function in tumor microenvironment. Our results revealed the presence of the IL-17/miR-192/IL-17Rs loop in MM cells, and implied that it may represent a new mechanism of carcinogenesis.

IL-17, produced locally in the tumor microenvironment, plays important roles in tumor immunity through ligand-receptor relationships. Inhibition of IL-17 significantly suppressed CD31, MMP9 and VEGF expression in tumor tissue and inhibited tumor growth [22]. Consistent with an oncogenic role of IL-17 in tumor development, tumor tissues have a higher frequency of IL-17 and IL-17^+^ T cells [23]. The IL-17R family comprises five receptor subunits, IL-17RA-IL-17RE, which form productive receptor complexes to mediate activation of signaling in inflammatory and tumor events [24]. By far, IL-17RA, which is believed the largest member of the family, is a common signaling subunit used by the other four ligands. Through a distinct pathway depend on ACT1, IL-17RA activation leads to culminating in the activation of NF-κB [25], MAPK [26] and PI3K [27] pathways which are usually associated with tumorigenesis. Our data is consistent with these previous studies, and more importantly, our results indicated that IL-17RE may pair with IL-17RA to participate in IL-17 induced tumorigenesis.

Since their initial discovery, miRNAs have been implicated in the regulation of cellular processes which are deregulated in tumors, including proliferation, apoptosis, differentiation, cell migration and invasion [13], [14]. Depending on cellular contexts and target genes that they regulate, miRNAs may function as tumor suppressors or oncogenes [28]. miR-192 is well known as one of tumor suppressors and plays a regulatory role in tumor progression [15]–[17]. Feng S et al. [29] reported that miR-192 was expressed at low levels in lung cancer samples and targeted the RB1 gene to inhibit cell proliferation and induce cell apoptosis in lung cancer cells. In colon cancer, miR-192 regulates cell cycle and cellular proliferation by targeting dihydrofolate reductase [30]. In MM, our gain of function assays demonstrated that re-expression of miR-192 markedly inhibited cell growth and metastasis capabilities, by suppressing IL-17R expression, indicating that miR-192 might be a promising therapeutic target for multiple types of cancer treatment, including MM. Moreover, miR-215 is closely related to miR-192 because they share virtually identical transcriptional profiles, as shown in Fig. 5A. The biological prediction and our validation experiments found there was a similar regulation between miR-215 and IL-17Rs. Since miR-192 and miR-215 are highly homologous, we focused on the function of miR-192 throughout the rest of this study.

The biogenesis of miRNAs starts from primary transcripts, primary miRNAs (pri-miRNAs), under the control of RNA polymerase II and conventional transcription factors. The pri-miRNAs of intergenic miRNAs are transcribed under the control of distinct promoters or their host-gene promoters [31]. The regulation of miR-192 is largely unclear until now. Here we show that miR-192 can be directly repressed by IL-17. It is known that IL-17 activation induced transcription factor NF-κB [25] and MAPK [26], thus we proposed that miR-192 might be controlled by an upstream promoter containing a conserved NF-κB or MAPK binding site. Besides, the tumorigenic effects of IL-17 involve induction of IL-6 production, which in turn activates oncogenic signal transducer and activator of Stat3, which plays essential roles in the pathogenesis of many cancers [32]. Using ChIP assay, we demonstrated IL-17 directly repressed miR-192 by recruiting transcription factor p65. P65 is a ubiquitous transcription factor present in almost all cell types and related to many biological processes such as cell growth, tumorigenesis, apoptosis and inflammation [33]. Over the years, constitutive activation of p65 has been found in multiple types of human tumor, including lymphoma, multiple myeloma, ovarian cancer, lung carcinoma, breast cancer, thyroid carcinoma, melanomas, bladder cancer, pancreatic cancer and breast cancer [33]. The protumorigenic role of p65 signaling in epithelial cells was also reported [34]. Here we found that p65 downregulated miR-192 expression by directly binding to the miR-192 promoter; the downregulation was abrogated when p65 was repressed. Thus, our study suggests that p65 may be a cause for IL-17-induced dysregulated miR-192 expression observed in MM.

In conclusion, we revealed the oncogenic effects of IL-17 in MM, and elucidated the potential mechanism by which IL-17 is implicated in MM progression. IL-17 can induce proliferation, migration and EMT in MM cells by directly repressing miR-192, and miR-192 in turn suppresses expressions of IL-17Rs. Our study provides the first evidence that the IL-17/miR-192/IL-17Rs axis is manifest in MM and might represent a useful prognostic marker and therapeutic target for MM.

## References

[1] Gupta GP, Massague J (2006) Cancer metastasis: building a framework. Cell 127:679–695. 17110329 10.1016/j.cell.2006.11.001

[2] Ludwig H, Pohl G, Osterborg A (2004) Anemia in multiple myeloma. Clin Adv Hematol Oncol 2:233–241. 16163188

[3] Ribatti D, Vacca A (2014) The role of inflammatory cells in angiogenesis in multiple myeloma. Adv Exp Med Biol 816:361–376. 24818730 10.1007/978-3-0348-0837-8_14

[4] Olechnowicz SW, Edwards CM (2014) Contributions of the host microenvironment to cancer-induced bone disease. Cancer Res 74:1625–1631. 24599133 10.1158/0008-5472.CAN-13-2645 PMC3966188

[5] Kryczek I, Wei S, Zou L, Altuwaijri S, Szeliga W, et al (2007) Cutting edge: Th17 and regulatory T cell dynamics and the regulation by IL-2 in the tumor microenvironment. J Immunol 178:6730–6733. 17513719 10.4049/jimmunol.178.11.6730

[6] Kirkham BW, Kavanaugh A, Reich K (2014) Interleukin-17A: a unique pathway in immune-mediated diseases: psoriasis, psoriatic arthritis and rheumatoid arthritis. Immunology 141:133–142. 23819583 10.1111/imm.12142 PMC3904234

[7] Iwakura Y, Nakae S, Saijo S, Ishigame H (2008) The roles of IL-17A in inflammatory immune responses and host defense against pathogens. Immunol Rev 226:57–79. 19161416 10.1111/j.1600-065X.2008.00699.x

[8] Omrane I, Baroudi O, Bougatef K, Mezlini A, Abidi A, et al (2014) Significant association between IL23R and IL17F polymorphisms and clinical features of colorectal cancer. Immunol Lett 158:189–194. 24440568 10.1016/j.imlet.2014.01.002

[9] Liu J, Duan Y, Cheng X, Chen X, Xie W, et al (2011) IL-17 is associated with poor prognosis and promotes angiogenesis via stimulating VEGF production of cancer cells in colorectal carcinoma. Biochem Biophys Res Commun 407:348–354. 21396350 10.1016/j.bbrc.2011.03.021

[10] Das Roy L, Pathangey LB, Tinder TL, Schettini JL, Gruber HE, et al (2009) Breast-cancer-associated metastasis is significantly increased in a model of autoimmune arthritis. Breast Cancer Res 11:R56. 19643025 10.1186/bcr2345 PMC2750117

[11] Song XN, Yang JZ, Sun LX, Meng JB, Zhang JQ, et al (2013) Expression levels of IL-27 and IL-17 in multiple myeloma patients: a higher ratio of IL-27:IL-17 in bone marrow was associated with a superior progression-free survival. Leuk Res 37:1094–1099. 23849453 10.1016/j.leukres.2013.06.022

[12] Prabhala RH, Pelluru D, Fulciniti M, Prabhala HK, Nanjappa P, et al (2010) Elevated IL-17 produced by TH17 cells promotes myeloma cell growth and inhibits immune function in multiple myeloma. Blood 115:5385–5392. 20395418 10.1182/blood-2009-10-246660 PMC2902136

[13] Dogini DB, Pascoal VD, Avansini SH, Vieira AS, Pereira TC, et al (2004) The new world of RNAs. Genet Mol Biol 37:285–293. 10.1590/s1415-47572014000200014 PMC3983583 24764762

[14] Ambros V (2004) The functions of animal microRNAs. Nature 431:350–355. 15372042 10.1038/nature02871

[15] Khella HW, Bakhet M, Allo G, Jewett MA, Girgis AH, et al (2013) miR-192, miR-194 and miR-215: a convergent microRNA network suppressing tumor progression in renal cell carcinoma. Carcinogenesis 34:2231–2239. 23715501 10.1093/carcin/bgt184

[16] Boni V, Bitarte N, Cristobal I, Zarate R, Rodriguez J, et al (2010) miR-192/miR-215 influence 5-fluorouracil resistance through cell cycle-mediated mechanisms complementary to its post-transcriptional thymidilate synthase regulation. Mol Cancer Ther 9:2265–2275. 20647341 10.1158/1535-7163.MCT-10-0061

[17] Pichiorri F, Suh SS, Rocci A, De Luca L, Taccioli C, et al (2010) Downregulation of p53-inducible microRNAs 192, 194, and 215 impairs the p53/MDM2 autoregulatory loop in multiple myeloma development. Cancer Cell 18:367–381. 20951946 10.1016/j.ccr.2010.09.005 PMC3561766

[18] Heinemeyer T, Wingender E, Reuter I, Hermjakob H, Kel AE, et al (1998) Databases on Transcriptional Regulation: TRANSFAC, TRRD, and COMPEL. Nucleic Acids Res 26:364–370. 10.1093/nar/26.1.362 PMC147251 9399875

[19] Umansky V, Sevko A (2013) Tumor microenvironment and myeloid-derived suppressor cells. Cancer Microenviron 6:169–177. 23242672 10.1007/s12307-012-0126-7 PMC3717060

[20] Huston A, Roodman GD (2006) Role of the microenvironment in multiple myeloma bone disease. Future Oncol 2:371–378. 16787117 10.2217/14796694.2.3.371

[21] Chang SH, Mirabolfathinejad SG, Katta H, Cumpian AM, Gong L, et al (2014) T helper 17 cells play a critical pathogenic role in lung cancer. Proc Natl Acad Sci U S A 111:5664–5669. 24706787 10.1073/pnas.1319051111 PMC3992670

[22] Li L, Boussiotis VA (2013) The role of IL-17-producing Foxp3^+^ CD4^+^ T cells in inflammatory bowel disease and colon cancer. Clin Immunol 148:246–253. 23773923 10.1016/j.clim.2013.05.003 PMC3808980

[23] Girardin A, McCall J, Black MA, Edwards F, Phillips V, et al (2013) Inflammatory and regulatory T cells contribute to a unique immune microenvironment in tumor tissue of colorectal cancer patients. Int J Cancer 132:1842–1850. 23002055 10.1002/ijc.27855

[24] Gaffen SL (2009) Structure and signalling in the IL-17 receptor family. Nat Rev Immunol 9:556–567. 19575028 10.1038/nri2586 PMC2821718

[25] Ruddy MJ, Wong GC, Liu XK, Yamamoto H, Kasayama S, et al (2004) Functional cooperation between interleukin17 and tumor necrosis factorα is mediated by CCAAT/enhancer binding protein family members. J Biol Chem 279:2559–2567. 14600152 10.1074/jbc.M308809200

[26] Shen F, Hu Z, Goswami J, Gaffen SL (2006) Identification of common transcriptional regulatory elements in interleukin-17 target genes. J Biol Chem 281:24138–24148. 16798734 10.1074/jbc.M604597200

[27] Huang F, Kao CY, Wachi S, Thai P, Ryu J, et al (2007) Requirement for both JAK-mediated PI3K signaling and ACT1/TRAF6/TAK1-dependent NF-κB activation by IL-17A in enhancing cytokine expression in human airway epithelial cells. J Immunol 179:6504–6513. 17982039 10.4049/jimmunol.179.10.6504

[28] Calin GA, Croce CM (2006) MicroRNA signatures in human cancers. Nat Rev Cancer 6:857–866. 17060945 10.1038/nrc1997

[29] Feng S, Cong S, Zhang X, Bao X, Wang W, et al (2011) MicroRNA-192 targeting retinoblastoma 1 inhibits cell proliferation and induces cell apoptosis in lung cancer cells. Nucleic Acids Res 39:6669–6678. 21511813 10.1093/nar/gkr232 PMC3159440

[30] Song B, Wang Y, Kudo K, Gavin EJ, Xi Y, et al (2008) miR-192 regulates dihydrofolate reductase and cellular proliferation through the p53-microRNA circuit. Clin Cancer Res 14:8080–8086. 19088023 10.1158/1078-0432.CCR-08-1422 PMC2653201

[31] Harada M, Luo X, Murohara T, Yang B, Dobrev D, et al (2014) MicroRNA regulation and cardiac calcium signaling: role in cardiac disease and therapeutic potential. Circ Res 114:689–705. 24526675 10.1161/CIRCRESAHA.114.301798

[32] Yu H, Jove R (2004) The STATs of cancer-new molecular targets come of age. Nature Reviews Cancer 4:97–105. 14964307 10.1038/nrc1275

[33] Yu LL, Yu HG, Yu JP, Luo HS, Xu XM, et al (2004) Nuclear factor-kappaB p65 (RelA) transcription factor is constitutively activated in human colorectal carcinoma tissue. World J Gastroenterol 10:3255–3260. 15484295 10.3748/wjg.v10.i22.3255 PMC4572290

[34] Stathopoulos GT, Sherrill TP, Cheng DS, Scoggins RM, Han W, et al (2007) Epithelial NF-kappaB activation promotes urethane-induced lung carcinogenesis. Proc Natl Acad Sci U S A 104:18514–18519. 18000061 10.1073/pnas.0705316104 PMC2141808

